# Supplementary material for: Genome-Wide Identification of the DOF Gene Family Involved in Fruitlet Abscission in Areca catechu L
Source: Int J Mol Sci. 2022 Oct 4;23(19):11768. doi: 10.3390/ijms231911768 (PMC9569674; doi:10.3390/ijms231911768)
Supplement: Supplementary file 1 [file ijms-23-11768-s001.zip › Table S2.pdf]

Table S2. Candidate motifs recognized by AcDOF4

Motif	Sequence	Function
GC-motif	+CGGGGGC	enhancer-like element involved in anoxic specific inducibility
	-GCCCCCG	
Unnamed	+CCGGCCC	
	-GGCCGGG	
Unnamed	+GCACGCC	
	-CGTGCGG	
Unnamed	+CCGGGGG	
	-GGCCC CC	
GC-motif	+CGGGGGG	enhancer-like element involved in anoxic specific inducibility
	-GCC CCCC	
Unnamed	+GGGGGGG	
	-CCCCCCC	
Unnamed	+CTGGTCC	
	-GACCAGG	
Unnamed	+CGCGGGG	
	-GCGCCCC	
Unnamed	+CGGGGCC	
	-GCCCC GG	
Unnamed	+GGGGGCC	
	-CCCCCGG	
Unnamed	+GGAGGGC	
	-CCTCCCG	
Unnamed	+GGGGGGC	
	-CCCCCCG	
Unnamed	+CCGGGGC	
	-GGCCCCG	
Sp1	+GGGGCGG	light responsive element
	-CCCCG CC	
Sp1	+CGGGCGG	light responsive element
	-GCCCGCC	
Unnamed	+CGCGGGC	
	-GCGCCCG	
E2Fb	+CGGCGGC	
	-GCCGCCG	
Unnamed	+CGCGCGG	
	-GCGCGCC	
Unnamed	+TAACGCC	
	-ATTGCGG	
Unnamed	+CGTCCGC	
	-GCAGGCG	
Unnamed	+TAGCTGC	
	-ATCGACG	

DRE core	+TGTCGGC
	-ACAGCCG
Unnamed	+CCCCGGG
	-GGGGCCC
Unnamed	+CGCGTGG
	-GCGCACC
Unnamed	+CGTCGGC
	-GCAGCCG
Unnamed	+GACCTGG
	-CTGGACC
Unnamed	+CGGGAGC
	-GCCCTCG
Unnamed	+TGTCTCT
	-ACAGAGA
DRE core	+CGGCTGC
	-GCCGACG
Unnamed	+CGCCGGG
	-GCGGCCC
Unnamed	+CGGCCCC
	-GCCGGGG
E2Fb	+TGGCGGC
	-ACCGCCG
Unnamed	+TGTCGG
	-ACAGCC
