# Supplementary material for: Genome-Wide Identification of the DOF Gene Family Involved in Fruitlet Abscission in Areca catechu L
Source: Int J Mol Sci. 2022 Oct 4;23(19):11768. doi: 10.3390/ijms231911768 (PMC9569674; doi:10.3390/ijms231911768)
Supplement: Supplementary file 1 [file ijms-23-11768-s001.zip › Table S3.pdf]

Table S3. Sequence information of the primers used in this study

Gene	Primer (5′ to 3′)
AcDOF1-F	agcagagaccttgcgttcaagc
AcDOF1-R	tggggtttcattgtctcggt
AcDOF2 -F	tggctcttctgatttggacaatgg
AcDOF2 -R	ggcagaatcttgtctggctt
AcDOF3 -F	agtgaaccagtgtgacccaa
AcDOF3 -R	atggcagaaccttatctggcttc
AcDOF4 -F	gatcagctgcataactccagcgat
AcDOF4 -R	gtaatactccctgtcgcagtcta
AcDOF8 -F	tcggtcccgaatagcatcccccct
AcDOF8 -R	agggcctggtagtcctccacct
AcDOF9 -F	cacagcagcatcatccacggt
AcDOF9 -R	gggaaagtcgttgaactcgggca
PG-F	acgacgtggtaagtttcggagcca
PG-R	ccatggaagctggcttggct
PE-F	cagaactgcaacattttcgccaga
PE-R	gaacgaaccgttctgcgcacc
EXP2-F	accgctctcttcaacaacgggct
EXP2-R	tgtcgttggggagggcgtagttc
PE/PEI-F	gccctccgaataaacgcagacctg
PE/PEI-R	gattactgcggcgttgccaa
LRX-F	gaacaatgggctcaactcttgcct
LRX-R	gagccacatccagctgctccaa
XTH7-F	cttgttcgggcgtgtgagca
XTH7-R	ggaccgtgtaaggatgtccactcc
PIN-F	cctacagtctgatgcggaaatc
PIN-R	gaagcaaagcttcttgatccctgt
CKX-F	gtgttggagctcgaggttgtcacc
CKX-R	ccatttcaccatgcgaggggca
NCED-F	cggccatcacttcttcgacgggga
NCED-R	gagctcaccgatagccttgggga
PP2C-F	ttgcatgcagccaatgttggtgac
PP2C-R	gcgacaactcacatatcccccaga
SABP2-F	gtccccaattcatgtcactcaagc
SABP2-R	gtagaccacatcaaccgatcca
NPR1-F	aggtggaggaatttttcgcgatcc

NPR1-R	cgtgcaaacgtcgggctcca
AcActin-F	attcaggtgcccggaggtcctctt
AcActin-R	gggaacatggttgatcccccacta
